# Comparison of dynamic tumor tracking error measurement methods for robotic radiosurgery

**DOI:** 10.1002/acm2.14093

**Published:** 2023-07-11

**Authors:** Kohei Okawa

**Affiliations:** ^1^ Faculty of Health Sciences Butsuryo College of Osaka Sakai Osaka Japan; ^2^ Department of Radiotherapy Quality Management Shinryoku Neurosurgical Clinic Yokohama CyberKnife Center Yokohama Japan

**Keywords:** CyberKnife, dynamic tumor tracking, tracking error

## Abstract

**Background:**

Dynamic tumor motion tracking is used in robotic radiosurgery for targets subject to respiratory motion, such as lung and liver cancers. Different methods of measuring tracking error have been reported, but the differences among these methods have not been studied, and the optimal method is unknown.

**Purpose:**

The purpose of this study was to assess and compare tracking errors encountered with individual patients using different evaluation methods for method optimization.

**Methods:**

We compared the beam's eye view (BEV), machine learning (ML), log (addition error: AE), and log (root sum square: RSS) methods. Log (AE) and log (RSS) were calculated from log files. These tracking errors were compared, and the optimal evaluation method was ascertained. A *t*‐test was performed to evaluate statistically significant differences. Here, the significance level was set at 5%.

**Results:**

The mean values of BEV, log (AE), log (RSS), and ML were 2.87, 3.91, 2.91, and 3.74 mm, respectively. The log (AE) and ML were higher than BEV (*p* < 0.001), and log (RSS) was equivalent to the BEV, suggesting that the log (RSS) calculated with the log file method can substitute for the BEV calculated with the BEV method. As RSS error calculation is simpler than BEV calculation, using it may improve clinical practice throughput.

**Conclusion:**

This study clarified differences among three tracking error evaluation methods for dynamic tumor tracking radiotherapy using a robotic radiosurgery system. The log (RSS) calculated by the log file method was found to be the best alternative to BEV method, as it can calculate tracking errors more easily than the BEV method.

## 1 INTRODUCTION

Robotic radiosurgery is performed using a radiation therapy device (CyberKnife®, Accuray Inc., Sunnyvale CA, USA) with a small linear accelerator mounted on a 6‐axis robotic arm.^1^, ^2^ Dynamic tumor motion tracking radiotherapy is used with this device in the treatment of targets subject to respiratory motion, such as lung and liver cancers.^3^, ^4^, ^5^ The accuracy of motion tracking depends on the characteristics of the patient's breathing.^6^, ^7^ In order to avoid tracking errors on a patient‐by‐patient basis, a margin is added to the clinical target volume, resulting in the planning target volume (PTV). According to Yang et al.,^8^ there are five types of errors to consider in dynamic tumor motion tracking of robotic radiotherapy: (1) segmentation uncertainty, (2) deformation uncertainty, (3) correlation uncertainty, (4) prediction uncertainty, and (5) targeting uncertainty. Of these, items (3), (4), and (5) are considered to affect the tracking error.

Several methods to quantify tracking errors have been proposed, including a method that analyzes log files (log file method),^9^ a method using an X‐ray beam or beam's eye view (BEV) method,^10^, ^11^, ^12^ and a method using machine learning (ML method).^13^ Using the log file method, (3) through (5) can be evaluated separately, as reported by Pepin et al.^9^ On the other hand, the BEV method includes all of (3) through (5) in a single assessment. It evaluates tracking errors by analyzing the beam's eye view and is considered the method that most closely reproduces the actual errors. The ML method is quite different from the above two methods in that it measures tracking error from respiration parameters. There may be some differences in the tracking errors due to differences in the measurement methods.

The purpose of this study was to verify the accuracy of tracking error measurements obtained by different evaluation methods and to propose an optimal method. Since it is important to know the relationships among the measurement methods, these relationships were clarified.

## 2 METHODS

### 2.1 The test plan

**FIGURE 1:**
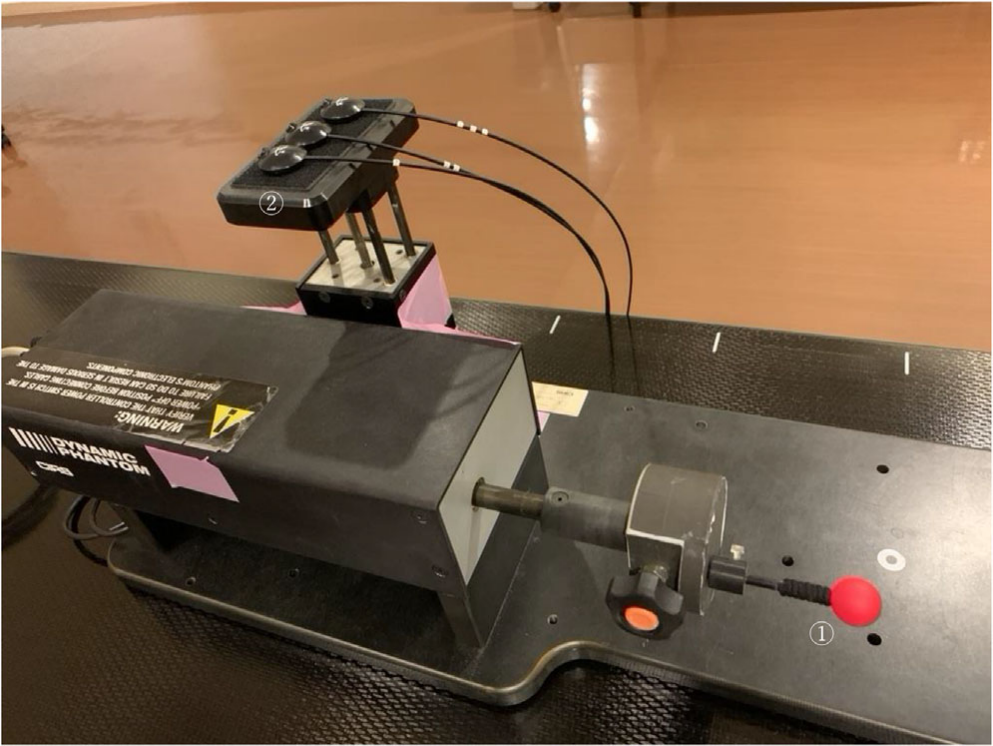
Dynamic motion phantom. Mechanism ① reproduces the movement of the target and ② reproduces the movement of the body surface.

**FIGURE 2:**
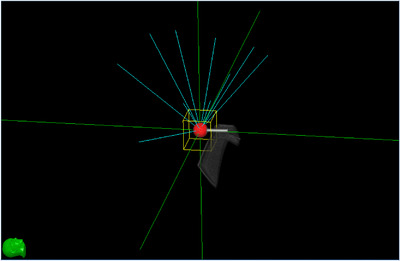
Schematic of the test plan. The red circle represents the target and the blue lines represent the radiation beam. The plan was created to irradiate from approximately 10 directions.

Image data of a dynamic motion phantom (CIRS Dynamic Thorax Phantom model 008A; Computerized Imaging Reference Systems, Inc. Norfolk, VA, USA) were acquired by computed tomography (CT) (SOMATOM Definition AS; Siemens, Munich, Germany). CT images were acquired at a tube voltage, current, and section thickness of 120 kV, 200 mA, and 1 mm, respectively. This phantom can reproduce the motion of the target and the body surface (Figure 1). The data were exported into a treatment planning system (MultiPlan®, Accuray), and a test plan was created to irradiate a simulated PTV in the phantom from 10 directions (Figure 2).

### 2.2 Respiratory data measurements

**FIGURE 3:**
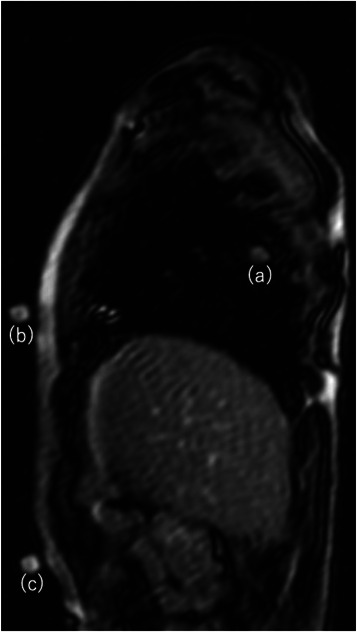
Cine MRI. (a) is the target and (b) and (c) are body surface markers. When the cine MRI was taken, multiple markers were attached, but when creating the waveform, a single marker was selected and the waveform was created.

The respiratory motion data used in this study were from 23 patients who underwent robotic radiosurgery at our institution. Fourteen were male and seven were female. Their ages ranged from 56 to 90 years (median: 78). The target site was the lung in 21 cases and adrenal gland in two. Respiratory data were acquired by cine magnetic resonance imaging (MRI) with a 1.5 T or 3 T MRI scanner (MAGNETOM Symphony® or MAGNETOM Skyra®, Siemens). A test tube containing contrast medium was affixed to the patient's chest (or abdominal) wall as an external marker. Noncontrast sagittal section images were acquired in a plane passing through the center of the tumor and the test tube (Figure 3) every 0.2–0.3 s. The sequence parameters were follows: echo time: 1.24–1.79 ms; repetition time: 4.3–139.2 ms; field of view: (208–291) × (400–500) mm; section thickness: 6–10 mm; flip angle: 8–20 degrees; band width: 450–500 Hz/pixel. Cine MR image analysis was performed with in‐house software to measure external markers and tumor motion. For the Cine MRI analysis, template matching based on the zero‐mean normalized cross‐correlation function was implemented, following Inoue et al.^10^ The clinical data acquired were used in this study. Informed consent was acquired for the use of the image data, which were anonymized.

### 2.3 Tracking error measurement

In this study, the BEV method reported by Inoue et al.,^10^ the log file method adopted from Pepin et al.,^9^ and ML method proposed by Okawa et al.,^13^ were used as tracking error measurement methods and compared.

#### 2.3.1 Tracking error measurement using the BEV method

Respiratory waveform data were registered to the dynamic motion phantom to reproduce the respiratory motion. Next, a small CCD camera was placed at the tip of the linac, and the test mentioned in **Section** **2** **A** was executed while acquiring video images, allowing the robotic arm to track the phantom. Tracking error was calculated by analyzing the video. The tracking error calculated here is defined as the BEV error.

#### 2.3.2 Tracking error measurement using the log file method

The log file method was based on the report of Pepin et al.^9^ Correlation uncertainty was defined as the value that covers 95% of the correlation errors acquired. The correlation uncertainty was calculated using the log file Model_point.log. Predictor.log and Modeler.log were then used to calculate prediction uncertainty. The value that covered 95% of the values calculated in the same way as the correlation uncertainty was used as the prediction uncertainty. The targeting uncertainty was uniformly set at 0.5, as reported by Pepin et al.^9^ Pepin et al. define the total tracking error as the addition of these values. We refer to this as “addition error.” As Yang et al.^8^ define it in terms of root sum square (RSS), the tracking error was also calculated in terms of RSS error. Based on Pepin et al.,^9^ each log file is described below:

1. The Modeler.log file contains the output of the correlation model (modeler points), giving the estimated tumor position at a given time.
2. The Predictor.log file contains the output of the prediction algorithm (predictor points) and the corresponding modeler points.
3. The Model_Points.log file contains the correlation error relating the modeler points to the x‐ray registration of tumor position.

#### 2.3.3 Tracking error measurement using the ML method

**FIGURE 4:**
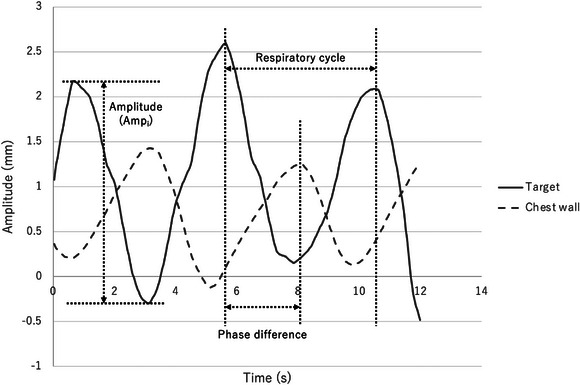
Definition of respiratory parameters. The amplitude was defined as the maximum to the minimum of the target waveform, and the respiratory cycle was defined as the respiratory cycle from the maximum to the next maximum. From the maxima of the target waveform to the maxima of the body surface waveform was the phase difference.

The ML method was reported by Okawa et al.^13^ Briefly, features for learning were calculated from the acquired respiratory waveforms. The features are amplitude variation (Amp_SD_), mean target velocity (TV_mean_), and phase difference (PD) between target and body surface markers. Target velocity was considered separately for the anterior‐posterior (AP) and craniocaudal (CC) directions. These were calculated by Equations (1) through (4) (Figure 4). The tracking error was then measured by a machine learning program.^13^ The tracking error acquired here is referred to as “ML error.”

(1) $$Amp_{mean}=\;\frac{1}{n}\;\sum_{i\;=\;1}^{n}Amp_{i}$$

(2) $$\;Amp_{SD}=\sqrt{\;\frac{1}{n}\;\sum_{i=1}^{n}{\left(Amp_{i}-\;Amp_{mean}\right)}^{2}}$$

where Amp_i_ is the amplitude of the target in one respiratory cycle (mm), *n* is the total number of amplitudes in the acquired waveform, and Amp_mean_ is the mean target amplitude (mm).

(3) $$\;TV_{i}=\;\frac{d}{t}$$

(4) $$\;TV_{mean}=\;\frac{1}{n}\;\sum_{i\;=\;1}^{n}TV_{i}$$

where TV_i_ is the target velocity in one respiratory cycle (mm/s), *d* is the distance traveled by the target in one respiratory cycle (mm), *t* is the duration of one respiratory cycle (s), and TV_mean_ is the mean target velocity (mm/s).

#### 2.3.4 Comparison of tracking error measurements

Because the BEV method evaluates tracking error by analyzing the video of the BEV, and is considered to be the method that most faithfully reproduces actual tracking error, the tracking error results acquired by each of the above methods were compared based on BEV error. A *t*‐test was performed to evaluate statistically significant differences. Here, the significance level was set at 5%.

#### 2.3.5 The evaluation of error concordance

Errors in BEV and correlation coefficients for each error were calculated. Additionally, regression analysis was conducted to calculate *p*‐values and coefficients of determination. Brandt‐Altman analysis was also performed, considering that regression analysis does not always indicate the degree of agreement, as reported.^14^, ^15^

## 3 RESULTS

### 3.1 Respiratory data

The results of the analysis of respiratory waveforms acquired from cine MRI are shown in Table 1. The median of amplitude variation was 0.72 (range: 0.11−3). The median velocities (mm/s) of the targets in the CC and AP directions were 2.61 (range: 0.37−7.10) and 1.29 (range: 0.22−2.13), respectively. The median of phase difference (s) was 0.14 (range: 0.07−0.89). The ML method was conducted using these data.

**TABLE 1:** Respiratory data.

	Target velocity (mm/s)		
Amplitude variation (mm)	CC direction	AP direction	Phase difference(s)
0.72 (0.11−3)	2.61 (0.37−7.10)	1.29 (0.22−2.13)	0.14 (0.07−0.89)

All values are expressed as median (range).

### 3.2 Final comparison of tracking error measurements

**FIGURE 5:**
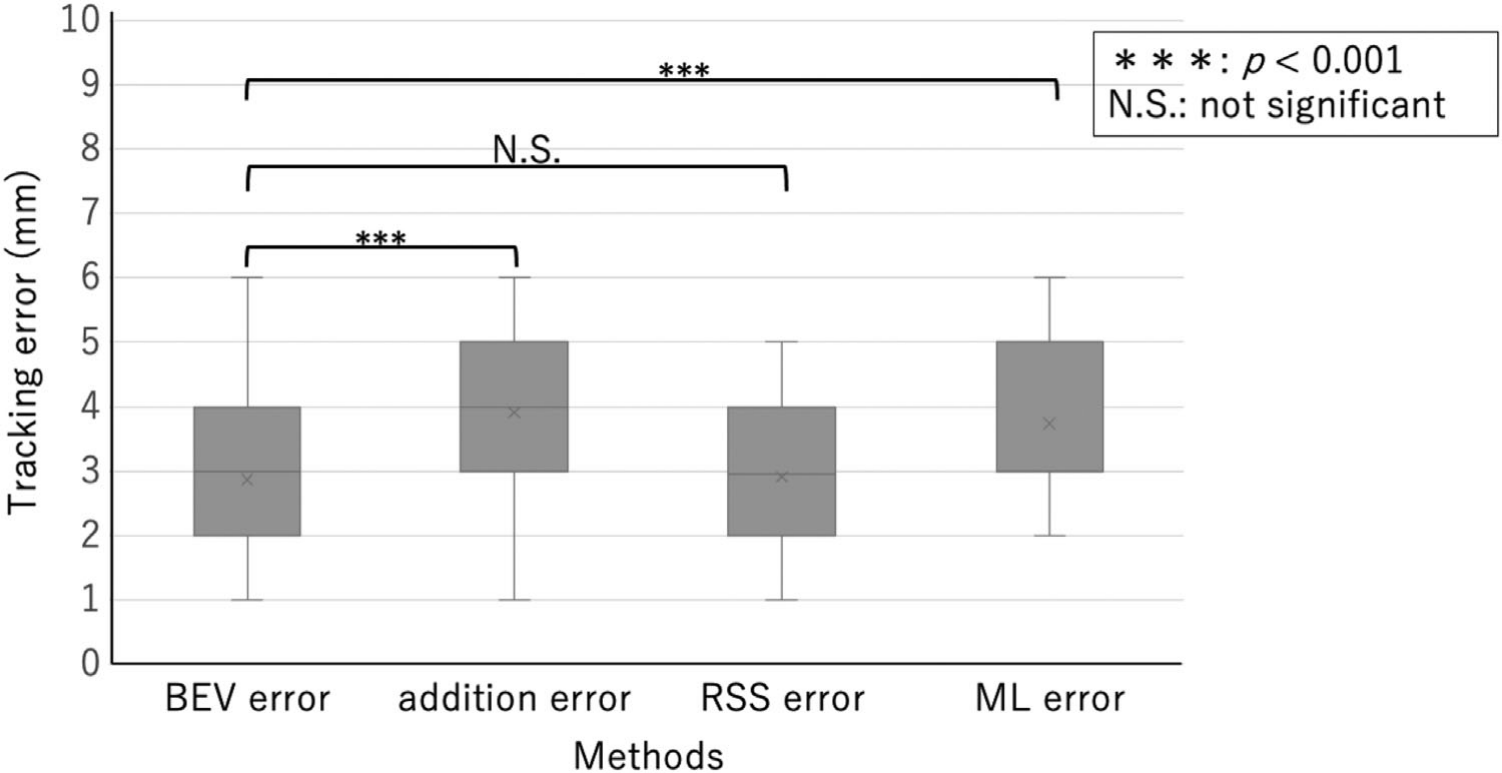
Box‐and‐whisker plot of tracking errors for each evaluation method, with significantly larger values for addition error and ML error compared to BEV error.

The results of the comparison of tracking errors are shown in Figure 5 The mean values of BEV error, addition error, RSS error, and ML error were 2.87, 3.91, 2.91, and 3.74 mm, respectively. Addition error and ML error were significantly larger than BEV error (*p* values from the *t*‐test were both < 0.001). RSS error was comparable to BEV error.

### 3.3 The evaluation of error concordance

**FIGURE 6:**
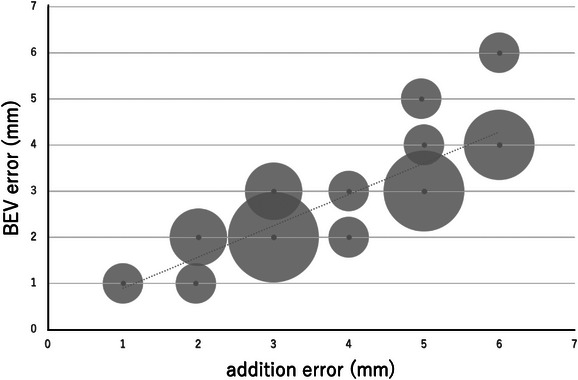
Scatterplot showing the relationship between BEV error and addition error. The size of the dots represents the frequency.

**FIGURE 7:**
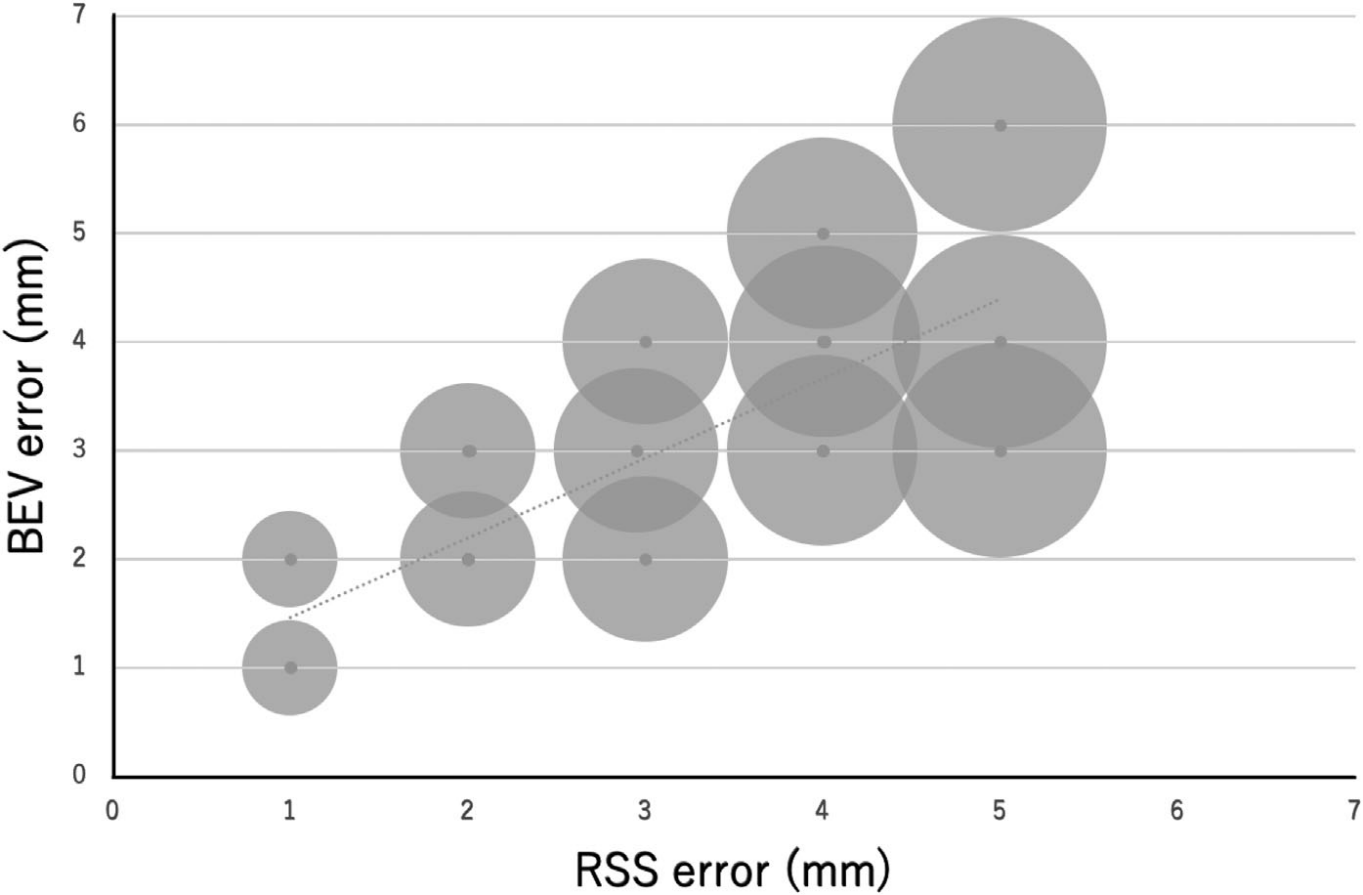
Scatterplot showing the relationship between BEV error and RSS error. The size of the dots represents the frequency.

**FIGURE 8:**
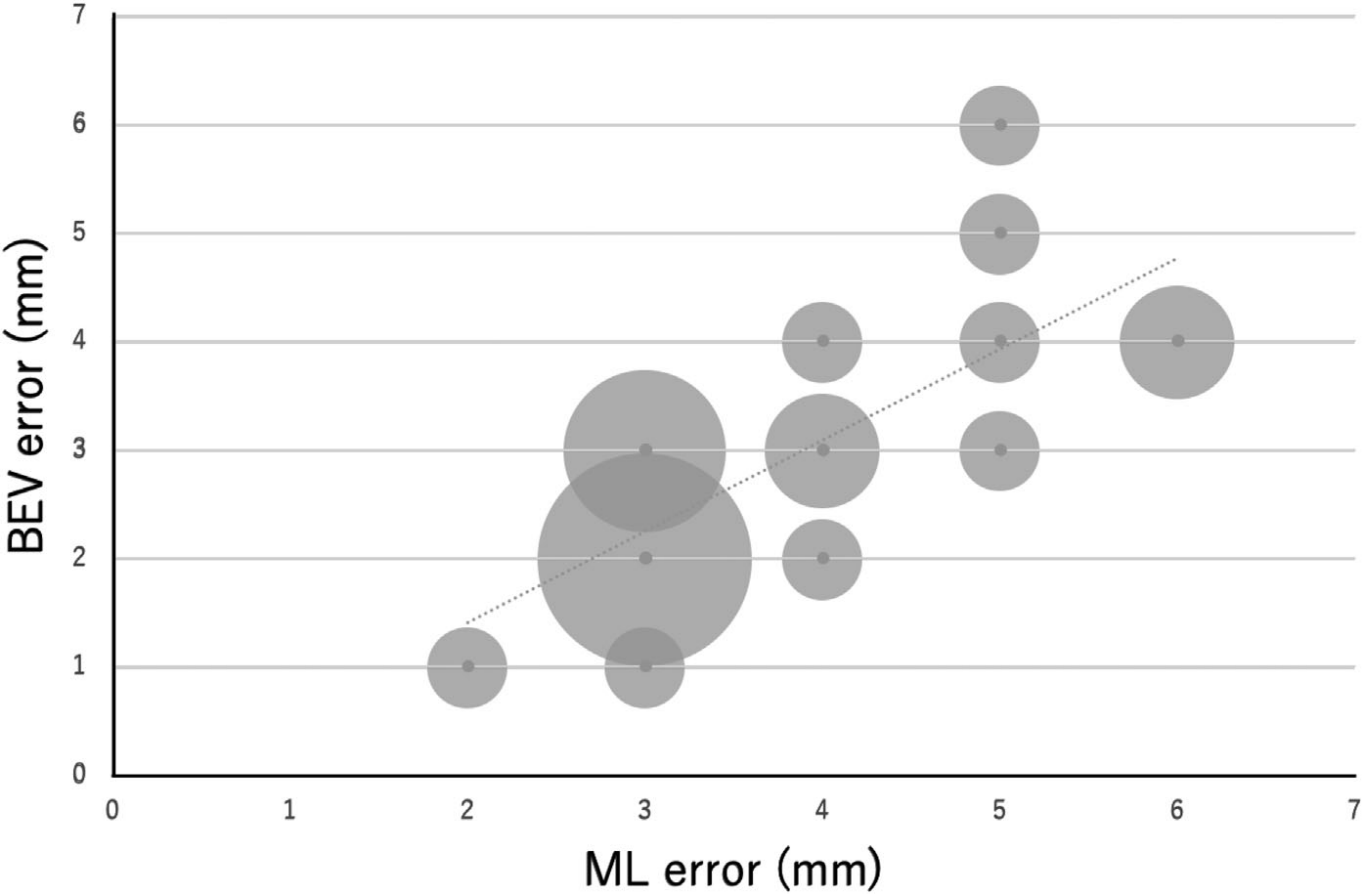
Scatterplot showing the relationship between BEV error and ML error. The size of the dots represents the frequency.

**FIGURE 9:**
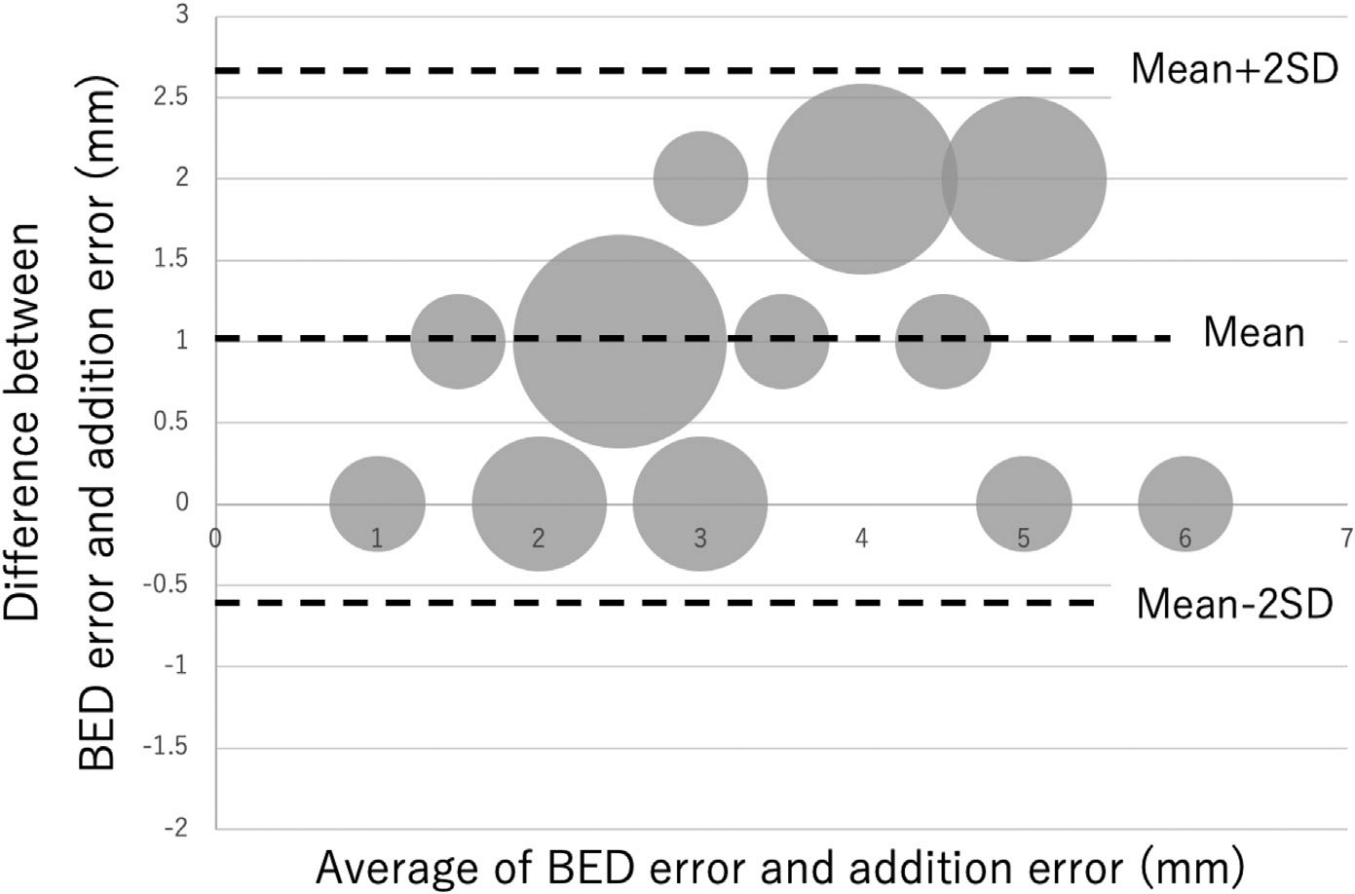
BA plot showing the relationship between BEV error and addition error. The size of the dots represents the frequency.

**FIGURE 10:**
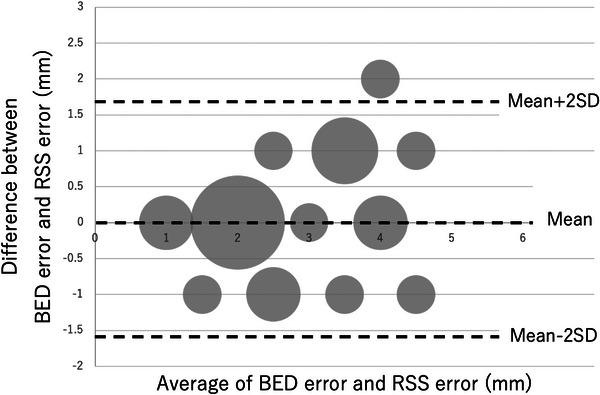
BA plot showing the relationship between BEV error and RSS error. The size of the dots represents the frequency.

**FIGURE 11:**
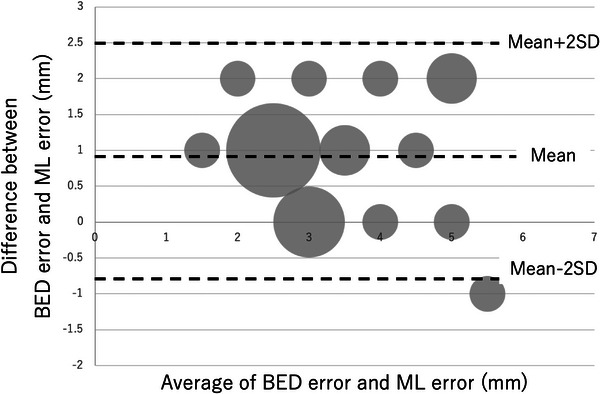
BA plot showing the relationship between BEV error and ML error. The size of the dots represents the frequency.

The results of the regression analysis are shown in Table 2. A graph of the relationship between each error and BEV error is also shown (Figures 6, 7, 8). Looking at these, all of these methods appear to be in good agreement with BEV. The results of the Brandt‐Altman analysis are shown in Figures 9, 10, 11. This analysis reveals more details. The differences between AD, ML, and RSS with respect to BEV were 1.0 ± 0.8(mm), 0.9 ± 0.8(mm), and 0 ± 0.8(mm), respectively. These graphs show that addition error and ML error overestimate slightly more than BEV error. And among the errors compared in this study, the RSS error was most consistent with the BEV error.

**TABLE 2:** Results of regression analysis of each error against BEV error.

	Regression coefficient	*p*‐value	R^2^
Addition error	0.676	<0.01	0.685
RSS error	0.734	<0.01	0.606
ML error	0.841	<0.01	0.552

## 4 DISCUSSION

This study clarifies the differences in tracking error measurement methods for dynamic tumor motion tracking radiotherapy using a robotic radiosurgery system. Considering each tracking error evaluation method, the BEV method was used as the standard in this study because it seemed to best reflect the actual tracking error. Compared to the BEV error acquired by the beam's eye view method, the addition error acquired by the log file method and the ML error acquired by the machine learning method were found to be larger. However, we believe that overestimating the tracking error is more acceptable than underestimating it, as this leads to adequate dose delivery to the target. On the other hand, the RSS error acquired by the log file method was equivalent to the BEV error. This result is more easily understood by the results of the Brandt–Altman analysis.

The log file method reveals that the calculated tracking error differs greatly due to differences in the calculation process, even when the same log file data was used. This is a point that needs to be considered when the log file method is used clinically. The fact that the RSS error was comparable to the BEV error compared to the addition error clearly indicates that the method of adding the squares as reported by Yang et al.^8^ is closer to the actual tracking error than the method of simply adding the elements of each error as reported by Pepin et al.^9^ when calculating the tracking error by the log file method.

As described in the report^13^ for the ML method, this system appears to be designed so that the measured tracking error is not less than the actual tracking error. Specifically, the system allows overestimation of up to 2 mm, validating the results of this study. As mentioned above, the BEV method seems to best reflect actual tracking errors, but all of the proposed methods have the disadvantage of being cumbersome and time‐consuming. Moreover, special phantoms and special analysis software are required. Medical physicists and radiotherapy technologists who are responsible for quality control of radiation therapy devices usually have to perform a large number of quality control test items, as indicated by the American Association of Physicists in Medicine task group.^16^ Working efficiently is very important in the clinical setting. Considering these points, the results of this study suggest that RSS error using the log file method may be an alternative to the BEV method, and may even allow for easier tracking error evaluation than the BEV method. This would be helpful for clinical medical physicists and radiotherapy technologists.

The study had several limitations. The first is that target movement is only evaluated in two dimensions. (AP and CC directions). However, Seppenwoolde et al.^17^ reported that target movement in the Right‐Left direction was smaller than in other directions. Inoue et al.^10^ also stated that the impact of the RL movement is limited. Based on the above, I believe the impact is small in this study as well. Second, only one movement of the body surface marker was evaluated. This impact cannot be determined in this study, and new efforts may be needed.

This study also clarified the relationship between the tracking error obtained by each method and the tracking error obtained by the BEV method. This allows us to understand the relationship between the tracking error obtained by each method and the reference tracking error, if the tracking error is obtained by a different method that is appropriate for the facility's situation. This would also be useful for clinical medical physicists and radiotechnologists.

## 5 CONCLUSION

This study clarified the differences in tracking error measurement methods for dynamic tumor tracking radiotherapy using a robotic radiosurgery system. Compared to the BEV error generated by the BEV method, the addition error resulting from the log file method and the ML error generated by the ML method were larger, while the RSS error resulting from the log file method was equivalent to the BEV error. In conclusion, it is clear that the RSS error generated by the log file method is the best alternative to the BEV error resulting from the BEV method. Considering the process of calculating the tracking error, the RSS error resulting from the log file method can calculate the tracking error more easily than the other conventional methods, and is expected to improve clinical throughput.

## AUTHOR CONTRIBUTION

Kohei Okawa was involved in every step of the process, from designing the study to writing the paper.

## CONFLICT OF INTEREST STATEMENT

The author has no relevant conflicts of interest to disclose.
